# A Morphological Hessian Based Approach for Retinal Blood Vessels Segmentation and Denoising Using Region Based Otsu Thresholding

**DOI:** 10.1371/journal.pone.0158996

**Published:** 2016-07-21

**Authors:** Khan BahadarKhan, Amir A Khaliq, Muhammad Shahid

**Affiliations:** 1 Department of Electronic Engineering, International Islamic University, Islamabad, Pakistan; 2 Department of Computer Engineering, CUST, Islamabad, Pakistan; Beijing University of Technology, CHINA

## Abstract

Diabetic Retinopathy (DR) harm retinal blood vessels in the eye causing visual deficiency. The appearance and structure of blood vessels in retinal images play an essential part in the diagnoses of an eye sicknesses. We proposed a less computational unsupervised automated technique with promising results for detection of retinal vasculature by using morphological hessian based approach and region based Otsu thresholding. Contrast Limited Adaptive Histogram Equalization (CLAHE) and morphological filters have been used for enhancement and to remove low frequency noise or geometrical objects, respectively. The hessian matrix and eigenvalues approach used has been in a modified form at two different scales to extract wide and thin vessel enhanced images separately. Otsu thresholding has been further applied in a novel way to classify vessel and non-vessel pixels from both enhanced images. Finally, postprocessing steps has been used to eliminate the unwanted region/segment, non-vessel pixels, disease abnormalities and noise, to obtain a final segmented image. The proposed technique has been analyzed on the openly accessible DRIVE (Digital Retinal Images for Vessel Extraction) and STARE (STructured Analysis of the REtina) databases along with the ground truth data that has been precisely marked by the experts.

## Introduction

DR is a severe disease and is one of the main source of visual impairment among adults aged 20–74 years in the United States [1]. The most common indications of DR include dilated retinal veins, hemorrhages, hard exudates and cotton wool spots [2]. Variation in features of vasculature of retinal images lead to serious diseases such as stroke, diabetes and cardiovascular diseases [3]. Consequently, an investigation of retinal vessel features can help with recognizing these progressions and permit the patient to make an action while the sickness is still in its initial stage. Automated investigation of the retinal blood vessels turned into a dynamic research because of its diagnostic and prognostic in the field of medical imaging.

Segmentation and review of retinal vasculature characteristics for example, tortuosity, normal or abnormal branching, shading and diameter as well as the optic disk morphology permits eye care experts and ophthalmologists to perform mass vision screening exams for early discovery of retinal ailments and treatment assessment. This could forestall and decrease vision debilitations, age-related diseases, and numerous cardiovascular ailments, and in addition diminish the expense of the screening [4, 5]. In manual assessment, segmentation and estimation accuracy also fluctuates relying upon nature of the retinal images, graders ability and experience. Moreover, manual segmentation and estimation procedures can take up to an hour for assessment of just a single eye. In this way, a completely automated framework extracting the vessel structures in retinal images could surely diminish the workload of eye clinicians.

This work consists of automated segmentation of vasculature of retinal images which can be used in diagnostic of various eye diseases. Different retinal vessel segmentation methodologies have been published and assessed in literature but they still need some improvement. Existing systems should be enhanced in terms of at least one of the following drawbacks. Firstly, lack of adaptive capabilities under varying image conditions may cause poor quality of segmentation such as under and over segmentations. Secondly, complex preprocessing and postprocessing operations used in different methods for extracting retinal images vessels caused high computational cost. Thirdly, human participation is required to choose area of interest, which demonstrates that the systems are not totally automatic. In conclusion, segmentation and assessment procedures themselves need a large computational endeavors.

## Related Works

In literature, many retinal segmentation methods are designed from the line detection methods, as vessel segmentation depends on line detection [6]. Generally, vessel segmentation methods consists of two steps: vessels enhancement and vessels classification. Some techniques may escape first step and use directly the second step. In the first step vessels are enhanced, noise and geometrical objects are removed. Chaudhuri et al. [7], first proposed matched filter to enhance and segment retinal vessels. Further improvements and similar techniques were proposed later on by various authors using threshold probing technique [8], double-sided thresholding [9] and the first order derivative of Gaussian image [10]. Matched filters application for segmentation, produced high quality results at the cost of long computational time. The quality of segmentation results mainly depend on the quality and size of the used vessel profile database. In [11], retinal blood vessels have been enhanced by using Gabor filter. This methodology has a great performance on normal retinal images. Lam and Yan [12], used laplacian operator and gradient vector fields to extract vessels. Staal et al. [13], proposed a framework depends on extraction of image ridges, which correspond roughly with vessel centerlines. Zana and Klein [14] and Mendonça and Campilho [15], used morphological filters to enhance vessels. Their proposed method showed better results than most of the existing techniques on the pathological retina.Martínez-Pérez et al. [16, 17], were also based on hessian matrix to extract multiscale feature for detection of vessels. In [18], vessel enhancement filter was designed on the base of hessian matrix.

After vessel enhancement, second step is the classification of pixels into vessel and non-vessel pixels. This second step is also known as vessel tracking and tracing [19]. Pixels intensities based classification is used to find a suitable threshold. Jiang and Mojon [20], performed adaptive local thresholding to extract vessels. In [21], Support Vector Machine (SVM) is used along with adaptive local thresholding to classify vessel and non-vessel pixels. Maritiner-perez et al. [17], extract information about vessel topology by using 1st and 2nd spatial derivatives of the intensity image. Zhou et al. [22], method is based on prior knowledge about retinal vessel characteristics coupled with matched filtering technique to detect the vessel structure. Al-Diri [23], utilized two pairs of contours to detect vessel boundary and sustain width of vessels. Fraz et al. [24], used first-order derivative of Gaussian filter to extract centerlines of retinal vessels before mathematical morphology to quantify vessels in retina. Generally, all vessel extraction methods can be classified into supervised segmentation [11–13, 25–31] and unsupervised segmentation [7, 9, 14–16, 23–24, 32–41] with the reference to the overall system design and structure.

Rest of the paper is arranged as follows: Section II, illustrate our suggested segmentation technique in detail. The preprocessing steps consist of CLAHE and morphological filters used for vessel enhancement and illumination corrections are discussed in detail. Further, hessian matrix and eigenvalues transformation are used in a modified form to compute the second derivative of the image at two different scales, for wide and thin vessels enhancement, separately. Otsu thresholding technique has been applied to classify vessel and non-vessel pixels. Finally, pixel count based thresholding has been applied to eliminate background noise, unwanted segments and erroneously detected vessel pixels. In section III, performance evaluation criteria is defined. Section IV, discussed the experimental Results. Finally, the proposed method is concluded in section V.

## Proposed Technique

**Fig 1:**
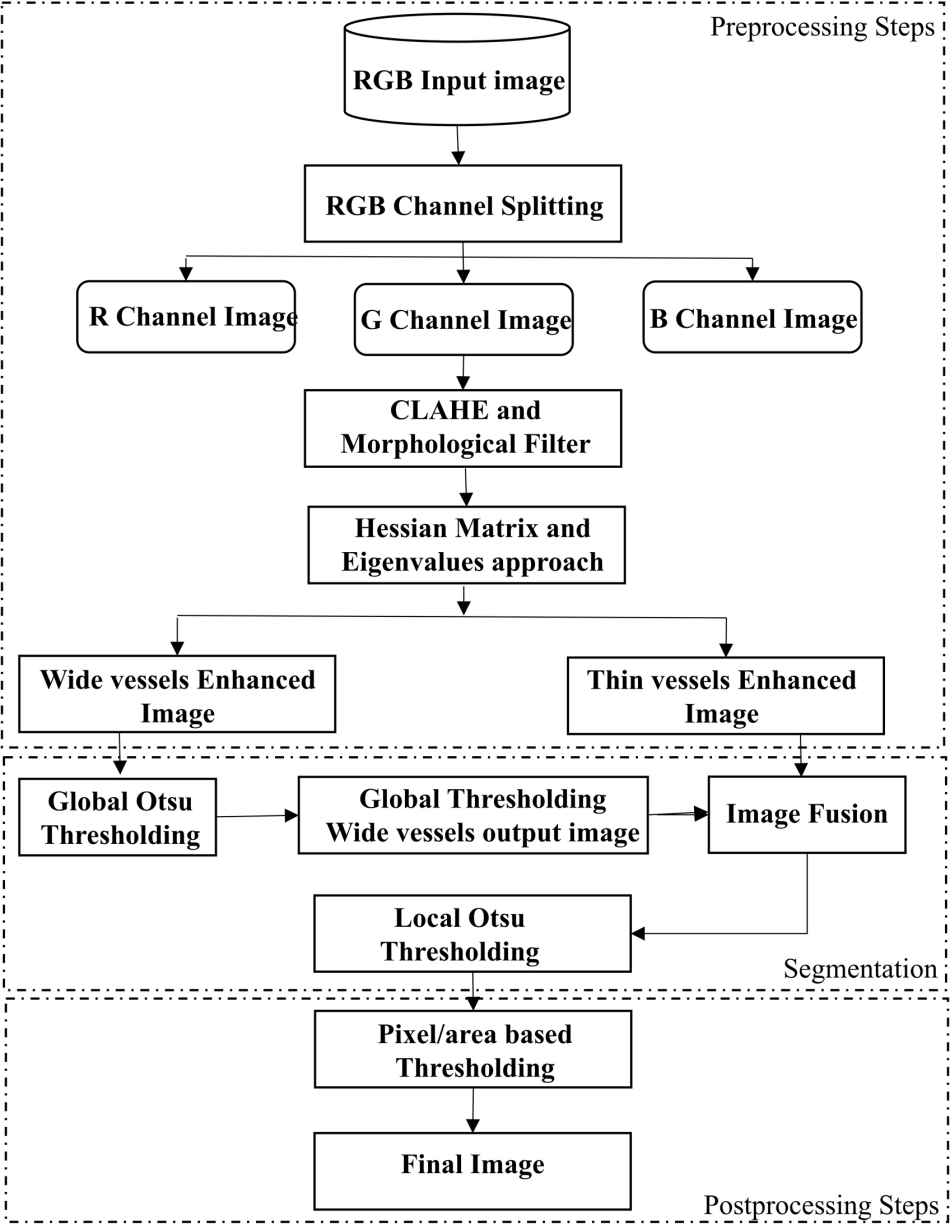
Flow chart of the proposed segmentation framework.

Fig 1 shows block diagram of our proposed segmentation framework. We extract green channel from input RGB retinal image for further processing.

The green band of input image is further analyzed by using following noteworthy steps:

- CLAHE and morphological filters have been used for vessel contrast enhancement and low frequency noise/geometrical objects removal respectively.
- Hessian matrix and eigenvalues transformation has been applied in a modified form at two different scales to extract wide and thin vessels enhanced images, separately.
- Global and local Otsu thresholding has been utilized in a modified way to classify vessel and non-vessel pixels from wide and thin vessel enhanced images, respectively.
- Postprocessing steps have been used for eliminating background noise, undesired segments and erroneously detected vessel pixels. We labelled vessel pixels ‘1’ and non-vessel pixels ‘0’ to obtain a final binary image.

### Contrast Limited Adaptive Histogram Equalization (CLAHE)

**Fig 2:**
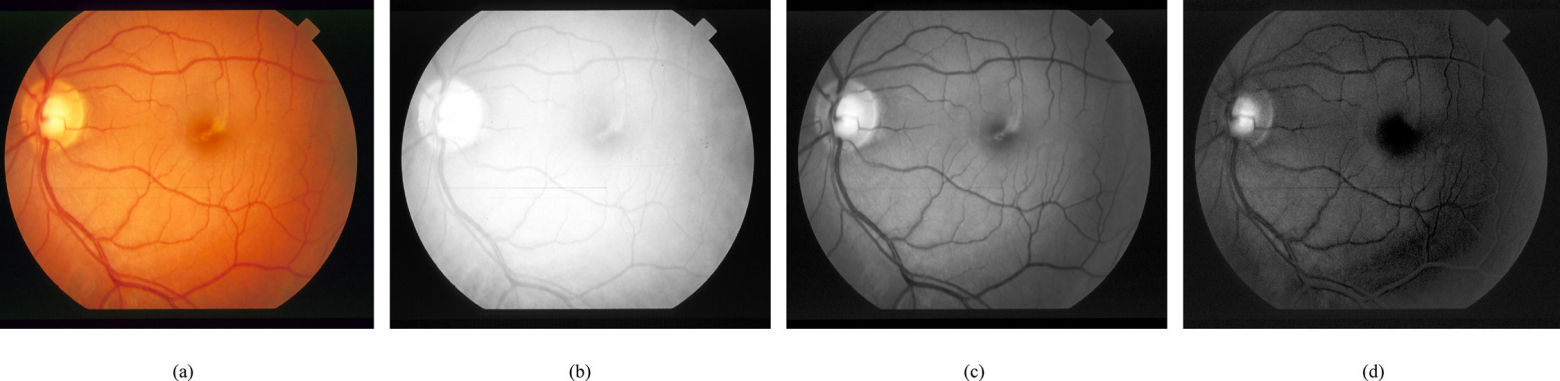
Retinal RGB image and its channels visual inspection. (a) RGB input image. (b) Red channel. (c) Green channel. (d) Blue channel.

We have used green channel of retinal image for analysis and segmentation of vessel structure. Fig 2 shows that in green channel, blood vessels seems more differentiated than the background as compared to red or blue channel. The DRIVE and the STARE datasets images are used for analysis and experiments of the proposed method.

Generally, histogram equalization techniques can acquire contrast improvement by stretching the gray level values of a low-contrast image. We used the CLAHE operator to achieve a local contrast enhanced retinal image. CLAHE used a user-defined value called clip limit to constrain enhancement by clipping the histogram [42]. The clipping level specifies noise level to be smoothen and contrast level to be enhanced in histogram. In our case, clip limit is set from 0 to 0.01.

### Morphological filters

Vessel enhancement based on morphological filters has already been published in literature [14]. Vessel structure appears in more prominent contrast than background intensity variations. However, a more local investigation of vessel intensities can indicate noteworthy changes that can adversely influence the whole vessel extraction process. To overcome such changes, we have proposed a morphological filter known as modified top-hat transform which has been applied on normalized green channel image. The thickest vessel width is set as a reference, which normally varies between 1 to 8 pixels covering all diameter ranges of vessels width for both image databases utilized as a part of our proposed scheme. Vessel diameter scale can be adapted according to image resolution variations.

We have utilized morphological top-hat transformation to find difference between the input and the opened image. Closed image followed by the opened image to obtain inverse image. Implementation of top-hat transformation has an issue of noise sensitivity, which cause that pixel values in an opened image are always less than or equal to the input ones; in such conditions, the subtracted image holds little intensity variations that can be found in the data. To solve this problem we adapted from [15], modified top-hat transform by introducing two new steps: a closing operator followed by the opening, without using any minimum operator and comparison.

The opening top-hat operator of an image ***I*** with structuring element ***S***_***o***_ is given by

$$T_{open}=I\circ S_{o}$$ (1)

The closing top-hat operator of an image ***I*** with structuring element ***S***_***c***_ is given by

$$T_{close}=I\cdot S_{c}$$ (2)

Modified top-hat transform adapted from [15] is given by

$$TopHat=I-(I\cdot S_{c})\;\circ \;S_{o}$$ (3)

Eq 3 shows our modified top-hat transform in which ***I*** is the input green channel image while ***S***_***c***_ and ***S***_***o***_ stand for the structuring elements for closing (⋅) and opening (∘) operators, respectively. In our case, we select disk type structuring elements for both opening and closing operator having radius eight pixels.

### Hessian Matrix and Eigenvalues based approach

We have applied hessian matrix and eigenvalues transformation in a new way after morphological filter to obtain enhanced images of wide and thin vessel. We have computed the second derivative of the image at two different scales for wide and thin vessel enhancement, separately. This isolation of wide and thin vessels has been achieved by using hessian matrix and eigenvalues based approach. The vessels having variable width are highlighted and based on the analysis of second order derivative at two different scales. The eigenvalues of hessian matrix and the difference between them have been used for further contrast enhancement and suppression of non-vasculature structure.

Hessian matrix of the directional image ***I***_***i***_ in the new coordinates ***Cx′y′*** is determined as [43]

$$H^{\prime }=\left[\begin{array}{c} h_{11}h_{12}\\ h_{21}h_{22} \end{array}\right]=\left[\begin{array}{c} \frac{\partial^{2}I_{i}}{\partial x^{\prime 2}}\frac{\partial^{2}I_{i}}{\partial x^{\prime }\partial y^{\prime }}\\ \frac{\partial^{2}I_{i}}{\partial y^{\prime }\partial x^{\prime }}\frac{\partial^{2}I_{i}}{\partial y^{\prime 2}} \end{array}\right]$$ (4)

where

$$\frac{\partial^{2}I_{i}}{\partial x^{\prime 2}}=\frac{\partial^{2}I_{i}}{\partial x^{2}}{cos}^{2}\theta_{i}+\frac{\partial^{2}I_{i}}{\partial x\partial y}\sin(2\theta_{i})+\frac{\partial^{2}I_{i}}{\partial y^{2}}{sin}^{2}\theta_{i}$$ (5)

$$\frac{\partial^{2}I_{i}}{\partial y^{\prime 2}}=\frac{\partial^{2}I_{i}}{\partial x^{2}}{sin}^{2}\theta_{i}-\frac{\partial^{2}I_{i}}{\partial x\partial y}\sin(2\theta_{i})+\frac{\partial^{2}I_{i}}{\partial y^{2}}{cos}^{2}\theta_{i}$$ (6)

$$\frac{\partial^{2}I_{i}}{\partial x^{\prime }\partial y^{\prime }}=\frac{\partial^{2}I_{i}}{\partial y^{\prime }\partial x^{\prime }}=-\frac{1}{2}\frac{\partial^{2}I_{i}}{\partial x^{2}}\sin(2\theta_{i})+\frac{\partial^{2}I_{i}}{\partial x\partial y}\cos(2\theta_{i})+\frac{1}{2}\frac{\partial^{2}I_{i}}{\partial y^{2}}\sin(2\theta_{i})$$ (7)

We have applied eigenvalues transformation on hessian matrix to obtain eigenvalues *λ*_1_ and *λ*_2_, while *σ* is used to define scale of vessel enhancement. The filter response will be optimum, if the scale *σ* matches the size of the vessel. In our case, *σ* ranges from 1 to 2.5 for vessels enhancement.

$${h^{\prime }}_{11}=\sigma^{2}\;*\;h_{11}$$ (8)

$${h^{\prime }}_{12}={h^{\prime }}_{21}=\sigma^{2}\;*\;h_{12}$$ (9)

$${h^{\prime }}_{22}=\sigma^{2}\;*\;h_{22}$$ (10)

$$\left[\lambda_{1}\lambda_{2}]=eign2img({h^{\prime }}_{11},{h^{\prime }}_{12},{h^{\prime }}_{22}\right)$$ (11)

Our method reduced the complexity and computation by taking only difference of *λ*_1_ and *λ*_2_ opposed to other existing methods using Frangi’s filter [18]. Difference images are obtained as *I*_*image*_ = *λ*_2_ − *λ*_1_ at two different scales having values 1 and 2.5 for *σ*.

**Fig 3:**
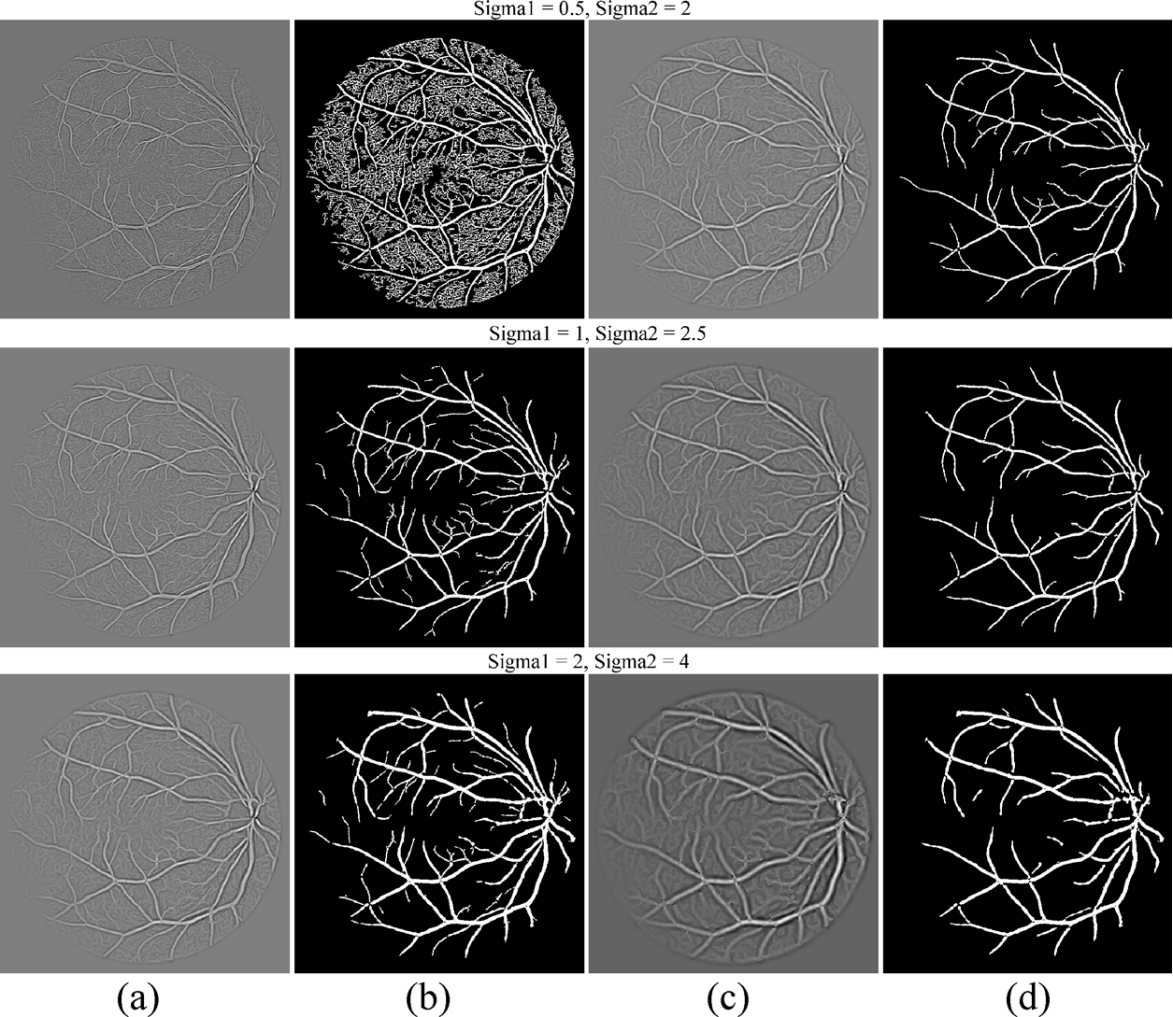
Comparison of the setting of parameter *σ* on different scales. (a) Thin vessel enhanced image. (b) Thin binary Image. (c) Thick vessel enhanced image. (d) Thick binary image.

We have tested the setting of parameter *σ* on different scalesas shown in Fig 3, which clearly indicates that setting of smaller scale increased False Positive Rate (FPR) considering background as a vessel pixels. In case of setting larger scale for *σ*, which is not able to detect thin vessel pixels decreasing the sensitivity of the proposed method.

### Otsu Method for Vessel Segmentation

We have applied Otsu’s approach [44] in a modified way to suppress the unwanted noise and geometrical objects based on vessel structure. Usually Otsu’s approach is used locally or globally on the entire image to find a threshold for classification of vessel and non-vessel pixels. Applying Otsu threshold on the whole image at once does not give fruitful results that’s why we have applied it separately on wide and thin vessel images. We have used global threshold on wide vessels enhanced image and fused its resultant image into thin vessel enhanced image. In this way both thin and thick vessels become more prominent. We obtained a single enhanced image on which further local thresholding has been applied. In local thresholding, we used vessel based thresholding which depends upon vessel locality to define a new threshold. We added some offset in the global threshold to suppress the noise more effectively for vessels in the neighborhood of wide vessels. For other regions away from wide vessels, we set lower threshold than the global by subtracting some offset from it to extract the small or thin vessels from the background having low intensity. Further, postprocessing steps have been applied to obtain final segmented image.

We used Otsu’s approach [44], to divide pixels of an image into two segments *S*_0_ and *S*_1_ (e.g., objects and background) at intensity level *t*, i.e, *S*_0_ = {0,1,2,…….,*t*} and *S*_1_ = {*t* + 1,*t* + 2,…….,*L* − 1}. As mentioned in [44], let *σ*^2^_*W*_,*σ*^2^_*B*_ and *σ*^2^_*T*_ be the within-class variance, between-class variance, and the total variance, respectively. We have minimized *σ*^2^_*W*_ to obtain optimal threshold. Following is the relation between different class variances.

$$\alpha =\frac{{\sigma^{2}}_{B}}{{\sigma^{2}}_{W}},\;\beta =\frac{{\sigma^{2}}_{B}}{{\sigma^{2}}_{T}},\;\gamma =\frac{{\sigma^{2}}_{T}}{{\sigma^{2}}_{W}}$$ (12)

The optimal threshold t* in our case is obtained by maximizing *α* and can be defined as

$$t^{*}=\underset{t\epsilon G}{\underbrace{ArgMax}}\;\alpha$$ (13)

where

$${\sigma^{2}}_{W}=\omega_{0}{\sigma^{2}}_{0}+\omega_{1}{\sigma^{2}}_{1}$$ (14)

$${\sigma^{2}}_{B}=\omega_{0}{\left(\mu_{0}-\mu_{T}\right)}^{2}+\omega_{1}{\left(\mu_{1}-\mu_{T}\right)}^{2}$$ (15)

$${\sigma^{2}}_{T}=\sum_{i=1}^{L}{\left(i-\mu_{T}\right)}^{2}P_{i}$$ (16)

$$\omega_{0}=\sum_{i=0}^{t}P_{i},\;\omega_{1}=1-\omega_{0},\;\mu_{1}=\frac{\mu_{T}-\mu_{t}}{1-\mu_{0}},\;\mu_{0}=\frac{\mu_{t}}{\omega_{0}}$$ (17)

$$\mu_{t}=\sum_{i=0}^{t}\;i\;P_{i},\;\mu_{T}=\sum_{i=0}^{L-1}iP_{i},\;G=\{0,1,2,\ldots \ldots .,L-1\}$$ (18)

where *n*_*i*_ is the total number of pixels with grey-level, *i* and *n* is the total number of pixels in the given image defined as $n=\sum_{i=0}^{L-1}n_{i}$. Probability of grey-level *P*_*i*_ is defined as $P_{i}=\frac{n_{i}}{n}$.

### Postprocessing Steps

We have used pixel/area based thresholding to eliminate unconnected non-vessel pixels. The segmentation results usually consist of some small isolated regions caused by noise, and these regions are sometimes wrongly detected as vessels. Based on the connectivity of the retinal vessels, we removed less than or equal to 30 unconnected pixels considered as a non-vessel or a part of the background noise.

## Performance Evaluation Criteria

We have processed retinal images from two publically available datasets: DRIVE [45] and STARE [8] for the performance evaluation of the proposed segmentation framework. These datasets consists of manual segmented retinal images by experts considered as a gold standard for comparison.

Accuracy (Acc), Sensitivity (Sn), Specificity (Sp), and the area under a Receiver Operating Characteristic (ROC) curve, also known as Area Under the Curve (AUC) are four commonly used parameters to compare the performance of the competing techniques [38]. Accuracy shows the overall segmentation performance. Sensitivity indicates effectiveness in detection of pixels with positive values: specificity measure the detection of pixels with negative values. These metrics are defined as follows:

$$Accuracy\;(Acc)=\frac{TP+TN}{TP+FP+TN+FN}$$ (19)

$$Sensitivity\;(Sn)=\frac{TP}{TP+FN}$$ (20)

$$Specificity\;(Sp)=\frac{TN}{TN+FP}$$ (21)

$$Area\;Under\;Curve\;(AUC)=\frac{Sn+Sp}{2}$$ (22)

where TP, TN, FP and FN represents the True Positive, True Negative, False Positive, and False Negative pixels, respectively.

## Experimental Results

**Fig 4:**
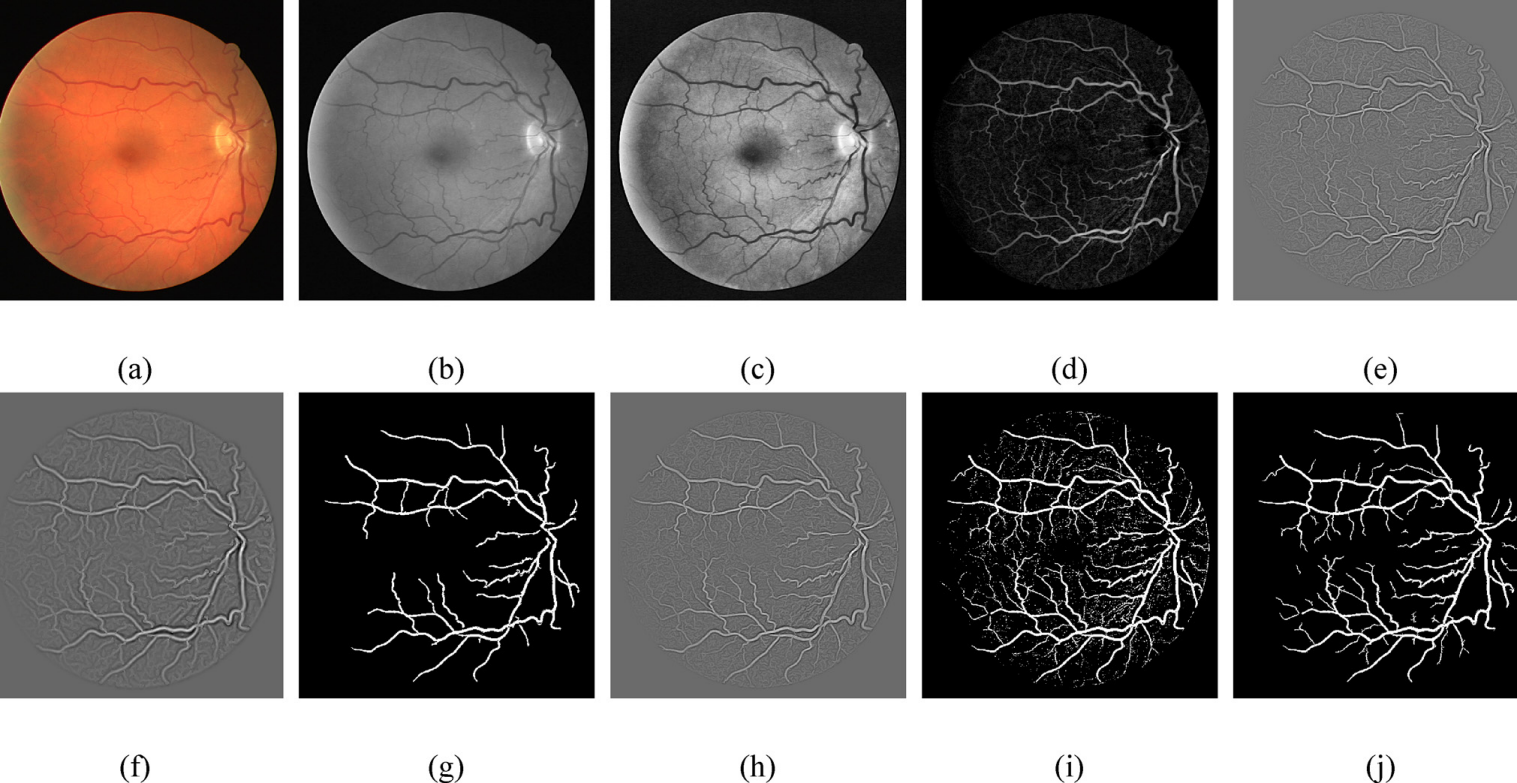
Proposed method main processing steps for retinal blood vessel segmentation. (a) RGB image from **DRIVE** database. (b) Green Channel. (c) CLAHE. (d) Morphological filters. (e) Thin vessel enhanced image. (f) Wide vessel enhanced image. (g) Otsu global thresholding output image. (h) Fused image of thin enhanced image and Otsu global thresholding output image. (i) Otsu local thresholding to enhance thin vessels (j) Postprocessed final binary image.

**Fig 5:**
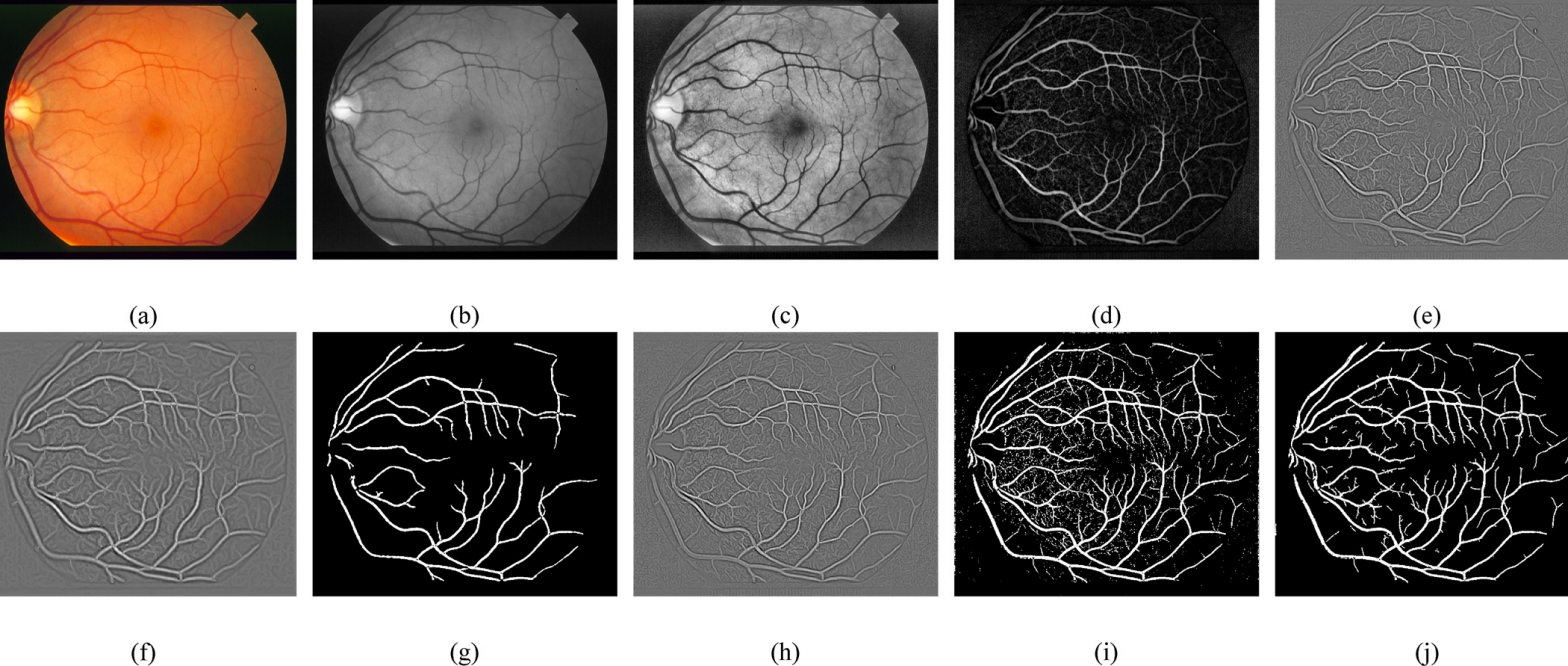
Proposed method main processing steps for retinal blood vessel segmentation. (a) RGB image from **STARE** database. (b) Green Channel. (c) CLAHE. (d) Morphological filters. (e) Thin vessel enhanced image. (f) Wide vessel enhanced image. (g) Otsu global thresholding output image. (h) Fused image of thin enhanced image and Otsu global thresholding output image. (i) Otsu local thresholding to enhance thin vessels (j) Postprocessed final binary image.

In this section, we have analyzed the performance of retinal vessel segmentation methods on the DRIVE [45] and the STARE [8] databases. The manually segmented images provided in these databases are used for better evaluation of the proposed framework. The DRIVE and the STARE datasets consist of 40 and 20 retinal images, respectively classified into two sets: the training set and the test set. For performance evaluation, the proposed framework has beenapplied on 20 test images of the DRIVE and the STARE datasets. All the experiments of our proposed framework were executed utilizing MATLAB 2013a on a DELL Vostro 1540 (2.53 GHz Intel Core i3 Processor, 4GB RAM). Visual inspection of retinal blood vessel segmentation with major processing stages of our proposed framework using the DRIVE and the STARE datasets are depicted in Figs 4 and 5, respectively.

**Fig 6:**
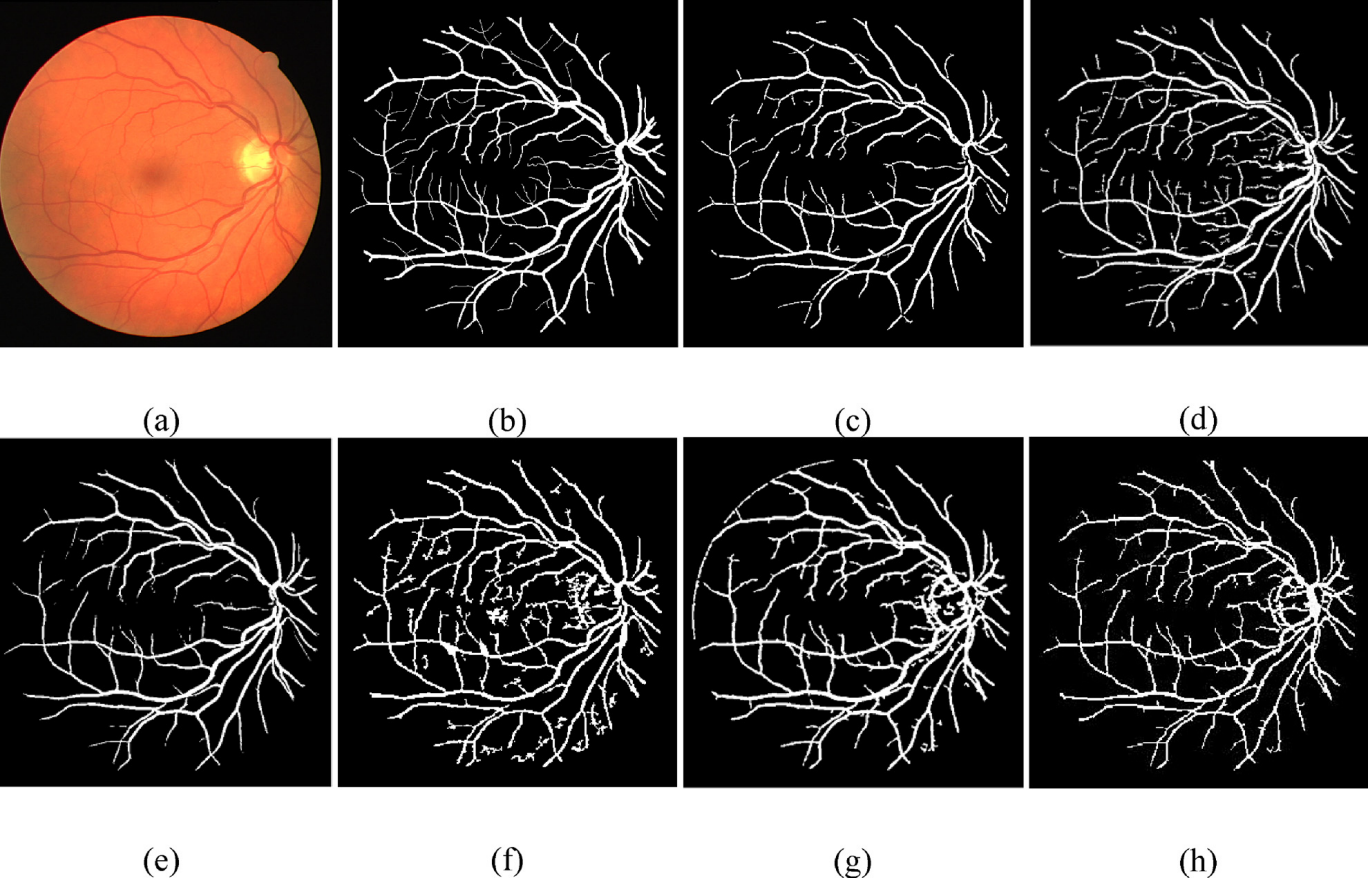
Visual inspection of different vessel segmentation methods using DRIVE database. (a) RGB input image. (b) Manual segmented image. (c) Proposed method final image. (d) Dai et al. [40]. (e) Azzopardi et al. [35]. (f) Bankhead et al. [30]. (g)Vlachos and Dermatas [41]. (h) Martinez-Perez et al. [17].

**Fig 7:**
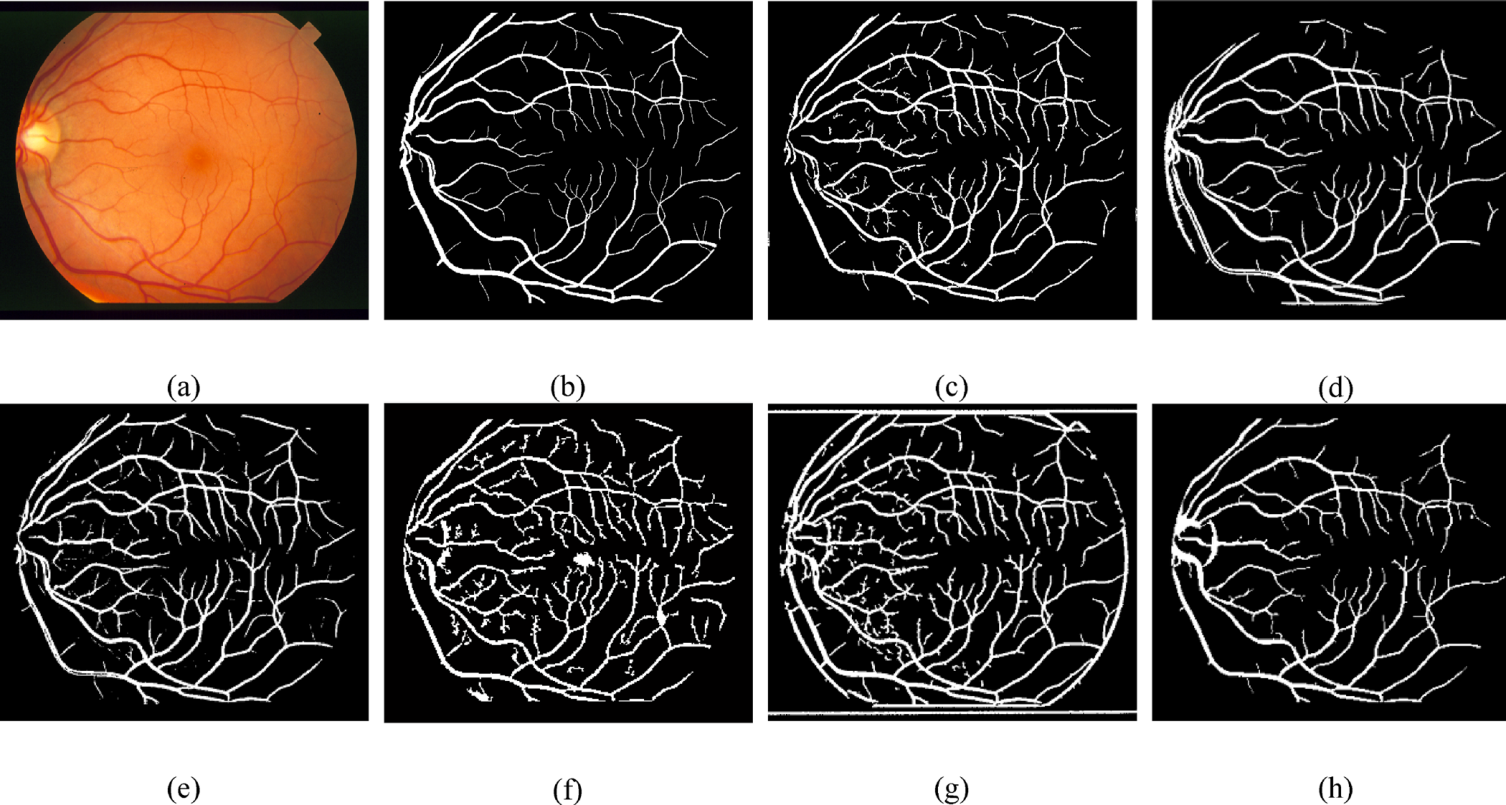
Visual inspection of different vessel segmentation methods using STARE database. (a) RGB input image. (b) Manual segmented image. (c) Proposed method final image. (d) Dai et al. [40]. (e) Azzopardi et al. [35]. (f) Bankhead et al. [30]. (g)Vlachos and Dermatas [41]. (h) Martinez-Perez et al. [17].

We have compared our visual results with Bankhead et al. [30] (S1 Link), Azzopardi et al. [35] (S2 Link), Dai et al. [40] (S3 Link), and Vlachos and Dermatas [41] (S4 Link) by running their source codes on the DRIVE and the STARE datasets shown in Figs 6 and 7, respectively. Images results of Martinez-Perez et al. [17] (S5 Link) were obtained from their website. To find whether a vessel is detected correctly or not, the final binary image has been compared to the corresponding manually segmented image.

### Average accuracy of the proposed method

First, we have calculated the average accuracy of 20 test images of the DRIVE dataset and 20 random images of the STARE dataset. The average accuracy indicates how to extract a binary image that matches the vessel images to a high degree. The accuracy is estimated by the ratio of the sum of the number of correctly classified foreground and background pixels, divided by the total number of pixels in the image. According to the results shown in Table 1, the average accuracy for the DRIVE dataset is 0.96075 and for the STARE dataset is 0.94585.

**Table 1:** Accuracy (Acc), Sensitivity (Sn) and Specificity (Sp) results of proposed method for 20 retinal images of the DRIVE and the STARE datasets.

Images	DRIVE			STARE		
	Acc	Sn	Sp	Acc	Sn	Sp
1	0.966	0.719	0.986	0.942	0.714	0.961
2	0.963	0.784	0.980	0.943	0.601	0.968
3	0.965	0.735	0.990	0.916	0.833	0.922
4	0.959	0.704	0.984	0.813	0.868	0.810
5	0.954	0.697	0.978	0.952	0.750	0.972
6	0.970	0.720	0.992	0.962	0.806	0.974
7	0.956	0.704	0.982	0.955	0.840	0.966
8	0.962	0.782	0.975	0.961	0.796	0.974
9	0.955	0.800	0.966	0.955	0.829	0.966
10	0.963	0.716	0.984	0.961	0.775	0.977
11	0.968	0.753	0.985	0.964	0.650	0.988
12	0.961	0.729	0.982	0.971	0.834	0.982
13	0.961	0.776	0.978	0.957	0.701	0.982
14	0.959	0.718	0.978	0.958	0.715	0.983
15	0.959	0.821	0.970	0.954	0.770	0.972
16	0.958	0.726	0.977	0.941	0.645	0.974
17	0.965	0.754	0.985	0.967	0.806	0.983
18	0.953	0.808	0.964	0.976	0.604	0.996
19	0.956	0.760	0.977	0.924	0.858	0.943
20	0.962	0.718	0.989	0.945	0.766	0.961
**Average**	**0.96075**	**0.7462**	**0.9801**	**0.94585**	**0.75805**	**0.9627**

### Proposed Otsu algorithm comparison with different techniques

**Fig 8:**
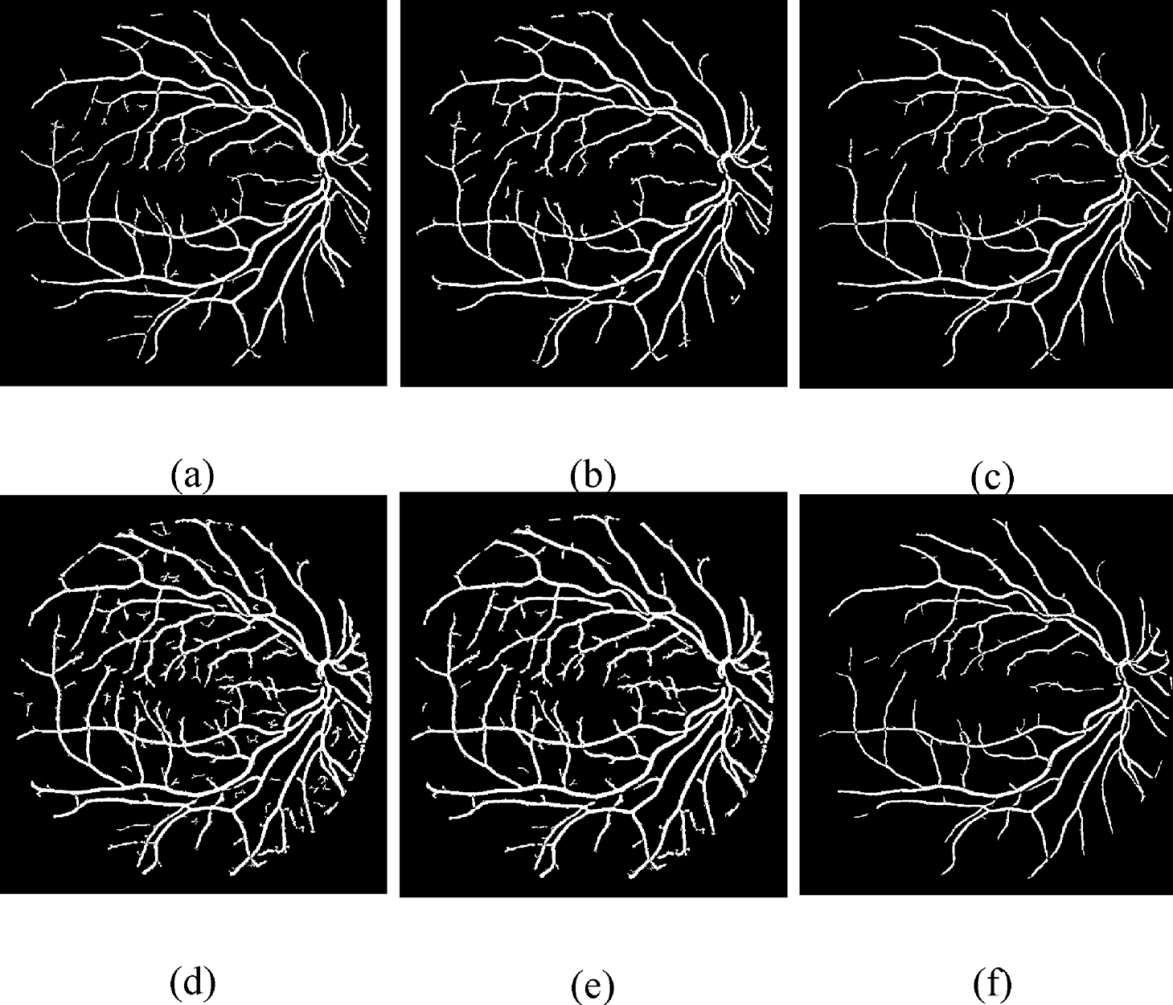
Visual results of different thresholding techniques. **(a) Proposed Otsu method.** (b) TILT. (c) K-means. (d) Moment-preserving thresholding. (e) Niblack local thresholding. (f) Fuzzy ISODATA algorithms.

We have compared the proposed Otsu approach [44] with current thresholding algorithms, Technique of Iterative Local Thresholding (TILT) [46], K-means [47], Moment-preserving thresholding [48], Niblack local thresholding [49] and fuzzy ISODATA algorithms [50]. The pictorial results on the DRIVE dataset have been displayed in Fig 8. Their performance in the term of accuracy, sensitivity, specificity and AUC has been tabulated in Table 2.

**Table 2:** Performance evaluation of different thresholding techniques with proposed Otsu method.

Method	DRIVE			
	AUC	Acc	Sn	Sp
Proposed Otsu [44]	0.882	0.963	0.784	0.980
TILT [46]	0.849	0.957	0.714	0.985
K-means [47]	0.812	0.956	0.631	0.993
Moment-preserving thresholding [48]	0.891	0.943	0.826	0.956
Niblack local thresholding [49]	0.888	0.946	0.815	0.961
Fuzzy ISODATA algorithms [50]	0.812	0.956	0.630	0.993

### Comparison with other techniques

In order to compare the efficiency of our proposed technique, we contrasted it with other existing vessel segmentation techniques on two commonly used databases: DRIVE [45] and STARE [8]. We have selected five latest supervised techniques [11–13,27, 31] and fourteen unsupervised techniques [7, 9, 14–16, 23–24, 30, 33, 35, 37–40]. The results are demonstrated in Table 3 which clearly indicates that our proposed framework is more efficient than many other methods.

**Table 3:** Performance evaluation of different retinal vessel segmentation techniques.

Method Type	Method	DRIVE			STARE		
		Acc	Sn	Sp	Acc	Sn	Sp
Unsupervised	Second observer	0.947	0.776	0.972	0.935	0.895	0.939
	Mendonça et al. [15]	0.945	0.734	0.976	0.944	0.699	0.973
	Martinez-Perez et al. [17]	0.934	0.725	0.965	0.941	0.751	0.955
	Palomera-Perez etal. [33]	0.922	0.660	0.961	0.924	0.779	0.940
	Zhang et al. [9]	0.938	0.712	0.973	0.948	0.717	0.975
	Fraz et al. [24]	0.943	0.715	0.976	0.944	0.731	0.968
	Bankhead etal. [30]	0.937	0.703	0.971	0.932	0.758	0.950
	Chaudhuri et al. [7]	0.877	0.336	—	—	—	—
	Zana and Klein [14]	0.938	0.697	—	—	—	—
	Al-Diri et al. [23]	—	0.728	0.955	—	0.752	0.968
	Azzopardi et al. [35]	0.944	0.766	0.970	0.949	0.772	0.970
	Mapayi et al. [37]	0.946	0.763	0.963	0.951	0.763	0.966
	Zhao et al. [38]	0.953	0.744	0.978	0.951	0.786	0.975
	Asad et al. [39]	—	—	—	0.934	0.748	0.954
	Dai et al. [40]	0.942	0.736	0.972	0.936	0.777	0.955
	**Proposed**	**0.961**	**0.746**	**0.980**	**0.946**	**0.758**	**0.963**
Supervised	Niemeijer et al. [31]	0.942	0.714	—	—	—	—
	Staal et al. [13]	0.944	0.719	0.977	0.952	0.697	0.981
	Ricci and Perfetti [25]	0.9595	—	—	0.9646	—	—
	Soares et al. [11]	0.946	0.723	0.976	0.946	0.723	0.976
	Lam et al. [12]	0.947	—	—	0.957	—	—
	Marin etal. [27]	0.945	0.706	0.980	0.952	0.694	0.981

“—” Shows that this content was not available.

Proposed framework shows highest results on the DRIVE images for both supervised and unsupervised methods, with Acc = 0.961, Sn = 0.746 and Sp = 0.980. Our proposed technique showed high efficiency in terms of sensitivity and accuracy among the unsupervised techniques on the STARE dataset. The specificity Sp = 0.963 which is also the highest value among the few unsupervised methods, and also behind the unsupervised techniques [9, 15, 24, 19, 35]. For supervised methods, accuracy is less 0.006 from [21, 27],0.0186 less from [25] and 0.011 behind [17], sensitivity is highest among all while specificity behind 0.018 than supervised methods [21, 27] and 0.013 less than [11].

**Fig 9:**
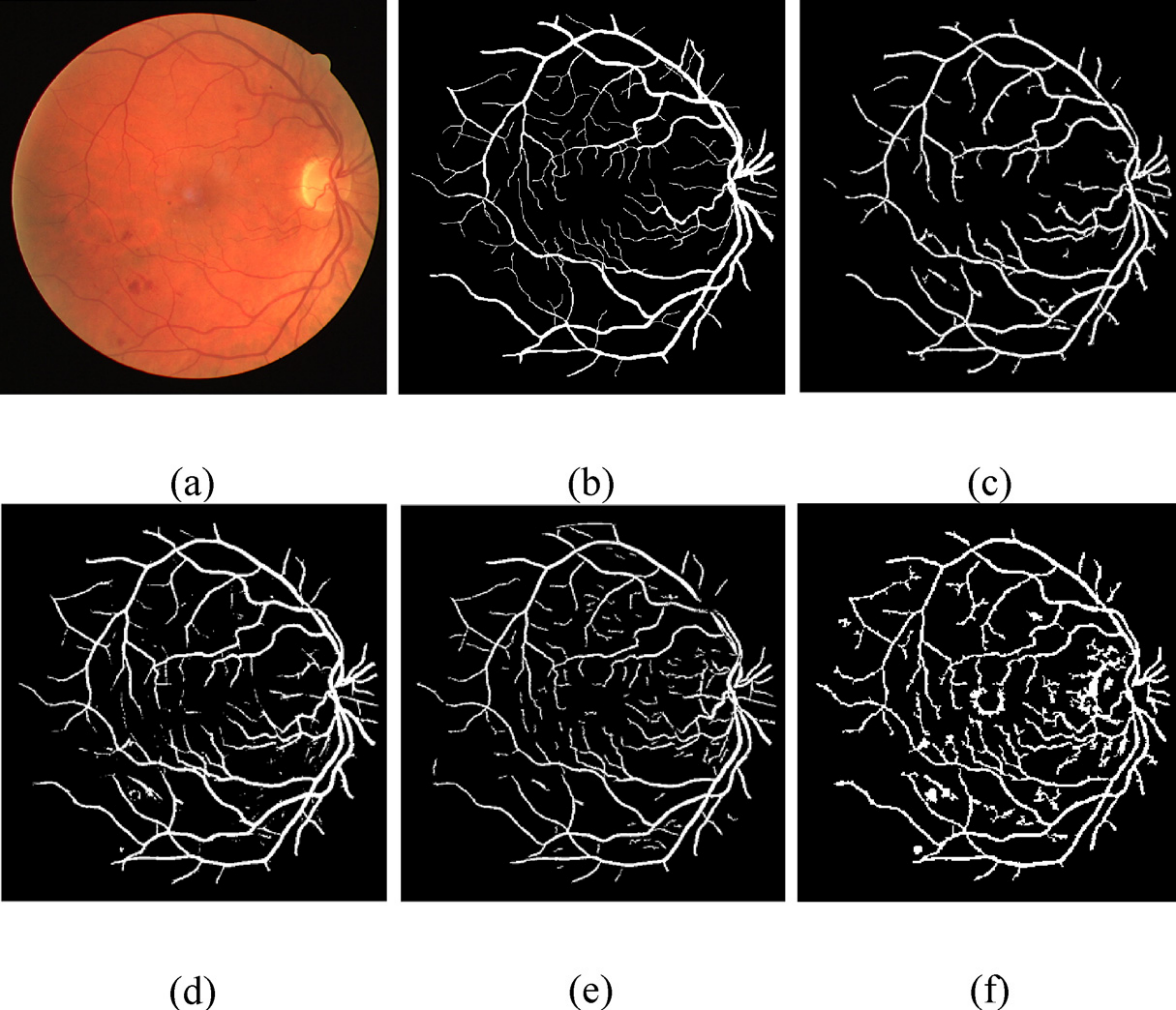
Pictorial results of different retinal blood vessel segmentation techniques on pathological image of DRIVE dataset. (a) RGB input image. (b) Manual segmented image. (c) Proposed method. (d) Azzopardi et al. [35]. (e) Dai et al. [40]. (f) Bankhead et al. [30].

**Fig 10:**
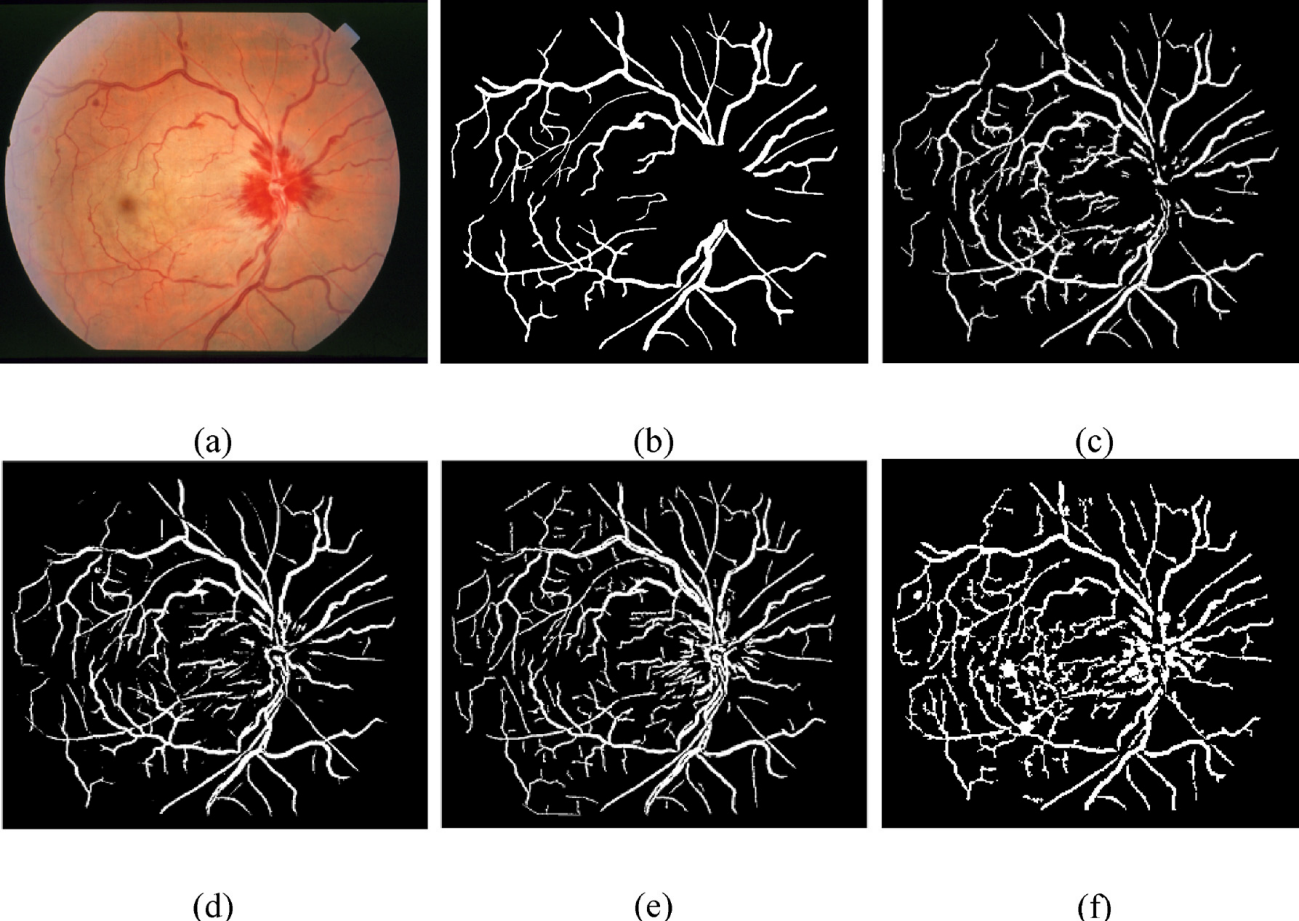
Pictorial results of different retinal blood vessel segmentation techniques on pathological image of STARE dataset. (a) RGB input image. (b) Manual segmented image. (c) Proposed method. (d) Azzopardi et al. [35]. (e) Dai et al. [40]. (f) Bankhead et al. [30].

One important factor of our proposed framework is to scale down the undesired segment, non-vessel pixels, erroneously detected segments and background noise that will frequently show up in the anomalous retinal images. For such pathological cases, we compared the performance of the proposed technique with various methods on the normal and abnormal images in the DRIVE and the STARE databases shown in Table 4, which evidently shows that for an abnormal cases, the proposed method achieve much better efficiency than Chaudhuri et al. [7], Mendonça and Campilho [15], Hoover et al. [8] and it records slightly better results than Soares et al. [11]. Figs 9 and 10 show visual appearance of an abnormal retinal images of the DRIVE and the STARE databases, respectively.

**Table 4:** Segmentation results comparison for normal versus abnormal cases of our proposed technique with different segmentation techniques.

Image Type	Method	DRIVE		
		TPR	FPR	Acc
Normal	Second observer	0.965	0.076	0.928
	Bankhead et al. [30]	0.752	0.047	0.942
	Mendonça et al. [15]	0.725	0.020	0.949
	Azzopardi et al. [35]	0.746	0.028	0.958
	Dai et al. [40]	0.738	0.036	0.953
	**Proposed**	**0.855**	**0.019**	**0.968**
Abnormal	Second observer	0.825	0.045	0.942
	Bankhead et al. [30]	0.754	0.043	0.939
	Mendonca et al. [15]	0.673	0.033	0.939
	Azzopardi et al. [35]	0.753	0.032	0.951
	Dai et al. [40]	0.782	0.035	0.948
	**Proposed**	**0.742**	**0.021**	**0.959**

Another important parameter is ROC curve, also known as AUC, it has the ability to reflect the trade-offs between the sensitivity and specificity. Note that an AUC of 0.50 means that the classification is equivalent to a pure random guess, and an AUC of 1.0 means that the classifier distinguishes class examples perfectly. The most frequently used performance measure extracted from the ROC curve is the value of the AUC which is 1 for an optimal system. The AUC achieved by the proposed method has been contrasted with existing segmentation techniques on the DRIVE and the STARE databases shown in Table 5. The proposed framework AUC results are highest than others on the DRIVE dataset except Mapayi et al. [37] and Ricci and Perfetti [25] which is 0.001 and 0.1003 little better than ours, respectively. On the STARE dataset, AUC of the proposed method is the highest among all, only [25], [38], [37] and [35] methods are 0.107, 0.02, 0.004 and 0.001 better than ours, respectively. Table 6, represents computation time comparison of various retinal vessel segmentation techniques. Computation time of Bankhead et al. [30], Azzopardi et al. [35], Dai et al. [40], and Vlachos and Dermatas [41] have been calculated by running their source codes on the PC (HP Intel Core i3 CPU, 2.53 GHz, 4 GB RAM), while computation time of Zhao et al. [38], Nguyen et al. [34], Mapayi et al. [37] and Asad et al. [39] have been collected from their published papers. The proposed framework is computationally very fast and efficient than other published methods.

**Table 5:** Performance comparison of AUC with existing techniques.

Method	AUC (DRIVE)	AUC (STARE)
Second observer	0.874	0.917
Azzopardi et al. [35]	0.862	0.862
Martinez-Perez et al. [17]	0.845	0.853
Palomera-Perez et al. [33]	0.811	0.860
Bankhead et al. [30]	0.837	0.854
Fraz et al. [24]	0.846	0.850
Ricci and Perfetti [25]	0.963	0.968
Zhao et al. [38]	0.861	0.881
Al-Diri et al. [23]	0.842	0.860
Marin et al [27]	0.843	0.838
Mapayi et al. [37]	0.864	0.865
**Proposed**	**0.863**	**0.861**

**Table 6:** Computation time comparison of various techniques.

Method	Processing Time	Computer Specifications	Software
**Proposed**	1.56 Sec	HP Intel Core i3 CPU, 2.53 GHz, 4 GB RAM	MATLAB
Bankhead et al. [30]	22.45 Sec		MATLAB
Azzopardi et al. [35]	11.83 Sec		MATLAB
Dai et al. [40]	1 min and 46 Sec		MATLAB
Vlachos et al.[41]	9.3 Sec		MATLAB
Zhao et al. [38]	4.6 Sec	HP Intel Core i3 CPU, 3.1 GHz, 8 GB RAM	MATLAB & C++
Mapayi et al. [37]	1.9 to 2.6 Sec	Intel Core i5 CPU, 2.30GHz, 4GB RAM.	MATLAB
Asad et al. [39]	2 mins and 45 Sec	Intel Core i3 CPU, 2.53 GHz, 3 GB RAM	MATLAB

## Conclusion

The automatic segmentation of blood vessels in retinal image is an important step in diagnosing causes of visual impairment. In our proposed framework, CLAHE and morphological filter has been used for vessel enhancement and low frequency noise/object removal along with hessian matrix and eigenvalues transformation to classify retinal image into wide and thin vessels enhanced images. Otsu thresholding has been utilized to extract vessel attributes and region properties based thresholding has been used set optimal threshold value to segregate vessel and non-vessel pixels. Proposed method has been applied to different databases like DRIVE and STARE and assessed based on performance measures such as sensitivity, specificity and accuracy. Further, our proposed method has been contrasted with different existing techniques to evaluate its efficiency and reliability. The proposed framework performs efficiently against noise and extract thin vessels. The proposed method is robust and computationally efficient.

## Supporting Information

S1 File Data used to test the algorithm. (ZIP)

S1 Link MATLAB implementation of Bankhead et al. [30] available at: http://journals.plos.org/plosone/article?id=10.1371/journal.pone.0032435. (ZIP)

S2 Link MATLAB source code of Azzopardi et al. [35] available at: http://www.mathworks.com/matlabcentral/fileexchange/49172-trainable-cosfire-filters-for-vessel-delineation-with-application-to-retinal- images. (ZIP)

S3 Link MATLAB implementation of Dai et al. [40] available at: http://journals.plos.org/plosone/article?id=10.1371/journal.pone.0127748. (ZIP)

S4 Link MATLAB source code of Vlachos and Dermatas [41] available at: https://matlabfreecode.wordpress.com/2013/02/27/detection-of-vessels-in-eye-retina-using-line-tracking-algorithm-with-matlab-code. (RAR)

S5 Link Images of Martinez-Perez et al. [17] are available at: http://turing.iimas.unam.mx/~elena/Projects/segmenta/VesselSegment.html. (RAR)
