# Correction: A Morphological Hessian Based Approach for Retinal Blood Vessels Segmentation and Denoising Using Region Based Otsu Thresholding

**DOI:** 10.1371/journal.pone.0162581

**Published:** 2016-09-01

**Authors:** Khan Bahadar Khan, Amir A Khaliq, Muhammad Shahid

The first author's name is spelled incorrectly. The correct name is: Khan Bahadar Khan. The correct citation is: Bahadar Khan K, A Khaliq A, Shahid M (2016) A Morphological Hessian Based Approach for Retinal Blood Vessels Segmentation and Denoising Using Region Based Otsu Thresholding. PLoS ONE 11(7): e0158996. doi:10.1371/journal.pone.0158996
